# NEVESIM: event-driven neural simulation framework with a Python interface

**DOI:** 10.3389/fninf.2014.00070

**Published:** 2014-08-14

**Authors:** Dejan Pecevski, David Kappel, Zeno Jonke

**Affiliations:** Institute for Theoretical Computer Science, Graz University of TechnologyGraz, Austria

**Keywords:** NEVESIM, neural simulator, spiking neurons, event-driven, Python

## Abstract

NEVESIM is a software package for event-driven simulation of networks of spiking neurons with a fast simulation core in C++, and a scripting user interface in the Python programming language. It supports simulation of heterogeneous networks with different types of neurons and synapses, and can be easily extended by the user with new neuron and synapse types. To enable heterogeneous networks and extensibility, NEVESIM is designed to decouple the simulation logic of communicating events (spikes) between the neurons at a network level from the implementation of the internal dynamics of individual neurons. In this paper we will present the simulation framework of NEVESIM, its concepts and features, as well as some aspects of the object-oriented design approaches and simulation strategies that were utilized to efficiently implement the concepts and functionalities of the framework. We will also give an overview of the Python user interface, its basic commands and constructs, and also discuss the benefits of integrating NEVESIM with Python. One of the valuable capabilities of the simulator is to simulate exactly and efficiently networks of stochastic spiking neurons from the recently developed theoretical framework of neural sampling. This functionality was implemented as an extension on top of the basic NEVESIM framework. Altogether, the intended purpose of the NEVESIM framework is to provide a basis for further extensions that support simulation of various neural network models incorporating different neuron and synapse types that can potentially also use different simulation strategies.

## 1. Introduction

Computer simulations of networks of spiking neurons are indispensable in modeling studies in computational neuroscience. Because of their importance, there have been many advancements in the development of simulation techniques for spiking neural systems. The growing body of work includes both innovations in novel algorithmic strategies that improve the efficiency, precision and scalability of the neural simulations, as well as innovations in design of software frameworks and software tools. There is a variety of useful neural simulation software tools that have emerged, and while they follow somewhat different approaches driven by a set of different requirements, there are also common principles in the algorithms and design that are shared among different simulators.

As any software project, the development process of a neural simulation software is faced with a challenge to design a software framework that satisfies best the defined requirements for the simulator. This entails finding the right concepts, and the relations between the concepts in the framework, that organize well the desired functionalities that are to be implemented. A well designed framework can make a big difference toward achieving at the same time generality, i.e., capability of simulating a broad range of different neural models, as well as flexibility and easy extensibility, while having minimum or no impact on the speed efficiency of the simulation. Therefore, it is of importance, in addition to publishing novel algorithms and data structures that improve the state of the art of simulation of certain types of neural systems, to also publish and share knowledge about software design approaches which enable many of these algorithms and models to seamlessly coexist organized in a single software framework. Indeed, there has been previous work (Diesmann and Gewaltig, 2001; Peck et al., 2003; Eppler et al., 2009; King et al., 2009; Pecevski et al., 2009; Djurfeldt et al., 2010) which reports on certain design aspects of the software framework and architecture of a particular neural simulation tool. As different neural simulators do have common points and common objectives in the development, developers of a new simulation tool can clearly benefit from this published work. They can learn from previous experience and improve their productiveness by borrowing already proven design ideas.

In this article we describe NEVESIM (Neural EVEnt-based SIMulator), an object-oriented framework for simulation of networks of spiking neurons developed in C++, with a Python interface. NEVESIM is intended for simulation of simple point neuron models, as well as neuron models composed of a few compartments. We will mainly focus on the software framework of the simulator, its concepts and constructs, as well as some useful design approaches that we adopted during the development of the framework.

The main motivation that lead to the development of NEVESIM was the need to be able to implement exactly and efficiently a certain class of neural models based on the recently developed theoretical framework of neural sampling (Buesing et al., 2011; Pecevski et al., 2011). The neural sampling framework formulated theoretical principles that enable construction of networks of stochastic spiking neurons which through their stochastic dynamics can perform probabilistic inference via Markov chain Monte Carlo (MCMC) sampling. A particular feature of the theoretically ideal neural sampling networks, which becomes relevant in context of their exact simulation in software^1^, is that the neurons communicate spikes between each other with a synaptic delay equal to 0. Although allowing small non-zero delay in the synaptic connections also leads to functional neural sampling models in most cases, for many purposes during our research work with neural sampling it was also desirable to be able to simulate the theoretically ideal neural sampling networks. For example, when analysing the performance properties of neural sampling, i.e., how fast the neural networks converge to a solution, it was important to include in the analysis the theoretically optimal models. It was also important to be able to simulate in a single simulation environment both the theoretically optimal, and the biologically more realistic models, in order to compare their performance. Furthermore, in addition to modeling and analyzing brain computations, the theory of neural sampling is also useful for implementing functional neural systems in neuromorphic hardware. Therefore it was of interest to perform software simulations of neural sampling networks that have some of the characteristics of the hardware, as an intermediate stage before porting the models to the hardware. As in some hardware systems the delays of the synaptic connections can be of an order of magnitude lower compared to biological neural systems, this was another example where simulations of networks with zero or very small delays (e.g., microseconds) were necessary. This entailed that, in order to enable simulation of networks with zero delay in the synaptic connections, we had to use an event-driven simulation algorithm with one centralized priority event-queue for the whole network to ensure correct causality. Other neural simulators in common use, like NEST (Gewaltig and Diesmann, 2007), have already developed frameworks and algorithms for efficient simulation of time-driven neuron models with precise spike times, as well as for embedding purely event-driven neuron model implementations in globally time-driven simulations (Hanuschkin et al., 2010). However, their simulation framewowk assumes a minimum delay *d*_min_ in the network connections which is larger than zero. This assumption brings several advantages, as the queue for handling events can be implemented with an efficient ring buffer data structure, and the simulation algorithm can be parallelized by having many event queues (in different processes on different machines) that are synchronized at time intervals equal to *d*_min_ (Morrison et al., 2005). Due to these advantages, other neural simulators have also adopted the same event handling strategy (Pecevski et al., 2009). Nevertheless, in the particular case of neural sampling networks this simulation framework was not applicable due to the specific requirements for simulating networks with zero delays. For these reasons, we chose to develop a new event-driven simulation framework to satisfy our simulation requirements. Moreover, for many probabilistic inference problems the corresponding neural sampling networks exhibit sparse connectivity (and hence sparse activity), i.e., they fall in the class of neural networks for which a globally event-driven simulation framework could be more efficient than a time-driven simulation framework with precise spike times. This is because with sparse activity and small delays the average number of input spikes per neuron per time-step can be smaller than 1 (Hanuschkin et al., 2010). Thus, apart from being able to handle correctly simulations of networks with zero delay connections, the event-driven framework had the potential of improving simulation efficiency for this type of sparse activity neural networks.

In addition to the algorithmic requirements, there was also a need for flexible configuration of the neural sampling networks that we used in our research work. For example, we needed to test neural networks with different shapes of EPSPs. We also wanted to analyse learning approaches, and for that purpose we needed to simulate networks that have plastic synapses with different plasticity rules as well as neurons with different intrinsic plasticity rules. Furthermore, for the purpose of learning there were additional mechanisms needed to be implemented, like changing the learning rate throughout the learning process, as well as injection of external currents in the neurons that represent the supervised signal given during learning. As several of these additional mechanisms required clock-driven simulation logic, it was also necessary to be able to embed clock-driven network elements in the simulation that encapsulate these mechanisms. The clock-driven network elements should update their state at equidistant time intervals, while the rest of the neural network is simulated in a purely event-driven way. Given these requirements, it was clear from the beginning that developing an ad-hoc specific simulator for neural sampling without any object-oriented framework would not suffice, as it would be cumbersome to maintain and extend it. Instead we needed to derive an event-driven simulation framework general enough to allow extensions in various directions, for various types of models. For this reason, one of the main objectives in the design was to support simulation of heterogeneous networks composed of different types of neurons and synapses, or other simulated network elements. Taking into account all the requirements, we set out to develop a conceptual software framework that contains just a few basic concepts, but is still able to integrate different simulation techniques and models and also exhibit good performance and extensibility.

An important component of every simulation tool is its user interface. The user interface should expose the functionalities of the simulator framework in a simple and intuitive way, and should allow easy and elegant expression of various operations on the neural models that are simulated. Because of their succinct syntax and flexibility, scripting languages are a natural choice for a simulator user interface (Hines and Carnevale, 1997; Bower and Beeman, 1998; Gewaltig and Diesmann, 2007). In particular, the Python programming language stands out as a scripting language that has been widely adopted by neural simulators and other neuroscience related software tools (Davison et al., 2009b,a). As it is a free, versatile, general-purpose, dynamic programming language with advanced features, and it has already proven to be very useful for many software packages in neuroscience, we also adopted Python as the best choice for a scripting user interface of NEVESIM. One of the additional advantages of Python is that there exist quite advanced wrapping tools that can be used to create a wrapper interface in Python for a software tool that is developed in another programming language (Abrahams and Grosse-Kunstleve, 2003; Beazley, 2003). In addition to presenting the overall framework of NEVESIM, we will also present in this paper aspects of the Python interface. In particular, we will cover some of its characteristic features and commands, presented through an example. We will also discuss how these features and commands relate to the conceptual framework of the simulator.

The contribution of this paper is twofold. The first and main contribution is the novel conceptual simulation framework together with its design principles and methods. One of the key design principles in the framework is construction of composite synapse and neuron models built out of more primitive components. This is enabled through the basic framework concept of network element which can be specialized to represent a single neuron, but also simpler functional parts within the neuron model. Another key feature of the framework is its capability of hybrid simulation of event-driven and time-driven network elements at the same time, achieved again by using the flexibility of the network element concept. The framework and its features bring many advantages, the most important ones being easy extensibility, better reusability of already implemented components that avoids code duplication, good organization of the code and easy code maintenance. A notable novel aspect of the framework is its ability to simulate configurable neural sampling models, which to the best of our knowledge was not addressed specifically in any previous work. Moreover, the applicability of the framework has a broader scope than neural sampling, as the simulator can be extended for simulation of various types of neural systems. The NEVESIM framework, its concepts and the design approaches are described in Section 2. Throughout this section there is also analysis, discussion and simple examples that motivate the framework constructs and the design choices made during development. In order to further demonstrate the useful features and advantages of the framework, in Section 3 we provide additional examples of how the framework concepts can be utilized to build specific neuron and synapse model implementations. We also describe in the same section how clock-driven simulation can be realized without introducing any additional framework concepts or mechanisms, which is another demonstration of the flexibility of the NEVESIM framework. The reader audience that would benefit mostly from the first contribution are developers of simulation tools that can adopt some of the design principles of the framework if they fit their requirements. Knowledge about the conceptual software framework would be certainly also valuable for researchers who want to extend NEVESIM for their own modeling and simulation needs. The second contribution of the paper is the presentation of the simulation software, where the main target reader audience are potential future users of NEVESIM. This part includes the description of the Python interface in Section 4, and the results of NEVESIM simulations of two example neural network models in Section 5 together with the code for implementing the model of the second example. In addition, in Section 5 we test the accuracy as well as the performance of the simulator in context of the model examples. The purpose of the second contribution is for the readers to get a first idea, impression of the capabilities, features, user-friendliness and performance of the simulation software, so that they can decide whether they want to use it for their research work.

## 2. The NEVESIM simulation framework

### 2.1. Basic concepts of the framework

#### 2.1.1. Network elements and event connections

The dynamics of mathematical models of networks of spiking neurons can be described through the equations governing the dynamics of each neuron and the communication of spikes, which represent point events, between the neurons. This allows one to make a first conceptual separation in the simulation engine in two parts: (i) handling of the communication of point events and (ii) numerical integration of the equations describing the dynamics of each neuron. As NEVESIM employs event-driven simulation strategy, the highest level of the simulation algorithm that drives the simulation forward in time is the emitting, scheduling and delivering of events. Furthermore, in NEVESIM the aim was to simulate heterogeneous networks of spiking neurons, where each neuron and synapse in the network can potentially be of different type and have an arbitrary different model dynamics. This was achieved by introducing an abstract concept of a network element from which all other neuron and synapse types will be derived. The concept of network element is implemented via the abstract class EvSimObject, and the simulation engine during execution of the simulation acts upon the network elements in the network only through the interface of this abstract class, without knowing the inner-workings of each individual network element. Thus, a simulated network in NEVESIM can be viewed as a network composed of interconnected network elements, which communicate to each other events in continuous time.

The concept of network element is quite general and is not restricted to just being a base concept for models of biological neurons and synapses. For example, input spike train generators and spike recorders are also implemented in NEVESIM as network elements. Moreover, in certain cases it is convenient to implement the computations of a sub-network of the biological neural network model with a more efficient non-neural algorithm, instead of instantiating a neural network for it. Additionally, in closed-loop experiments when the neural model needs to be coupled with an external environment, the environment can be implemented as a network element which is connected to the neural network via spiking connections in both directions. All these various cases of different network elements indicated the necessity for a network element to have maximum flexibility in forming incoming and outgoing connections with other network elements. This is accomplished in NEVESIM by allowing a network element to have multiple input and multiple output ports, through which it can receive or emit events.

The semantics of ports of the network elements can be summarized through the following definitions. Every instantiated network element in a NEVESIM network has an ID which uniquely identifies it within the network. The network element ID is a separate C++ data type, currently set to be equal to unsigned integer, which for most computer architectures has width of 32-bits^2^. An output port of a network element is identified by the ID of the network element it belongs to, and by the port number which is an integer value. The output ports that belong to a single network element are enumerated consecutively from 0 to the number of output ports minus 1. The same is true for the input ports. An event connection is defined through the quintuple (src_id, src_port, dest_id, dest_port, delay_time), meaning that events originating from the output port src_port of the network element with ID src_id are communicated to the input port dest_port of the network element with ID dest_id with a delay delay_time. The delay delay_time is a non-negative real number (can be also 0) specifying the delay in seconds.

#### 2.1.2. Coupling of network elements

Apart from interaction between the network elements via events, in NEVESIM there can exist also, what we call in the NEVESIM framework, coupling between the network elements. Two network elements A and B are considered to be coupled when the C++ object of A accesses during the simulation the C++ object of B either through invocation of a method of the C++ object, or by directly accessing one of its member variables. This is convenient and desirable in many cases, in particular when we want to have a processing node in the network (e.g., neuron) composed of many network elements. For example, the dynamics of certain point models of biological neurons can be decomposed into the dynamics of each of its input synapses and the dynamics of its membrane potential together with the spike generation mechanism. Thus, instead of having a neuron implemented as a single network element, in NEVESIM each synapse can be a separate network element which is coupled to its post-synaptic neuron (another network element). Furthermore, in synapses that exhibit synaptic plasticity one can implement the plasticity mechanisms in a separate network element and couple it to the network element implementing the synaptic dynamics. There are numerous other use cases where one can use multiple coupled network elements to implement a certain synapse or neuron model. We will present examples of coupled network elements on concrete implementations later in this section and in Section 3.

We distinguish between one-way and two-way coupling of network elements. If the network element A accesses methods or member variables of the network element B, and B does not access anything from A, then we call this one-way coupling from A to B. If the network element B also accesses A, then we have two-way coupling.

The approach to use composition of coupled network elements is not a novel idea, actually it follows the same principles of a well established design approach in object-oriented software design called object composition (Gamma et al., 1994). Thus, the advantages of using object composition that have been identified in software design in general, apply also to the case of coupled network elements in NEVESIM. One salient advantage of the approach is that it enables greater reusability of the network elements. Namely, one can break down the algorithmic logic of the neuron models (or also other processing nodes in the network) into well defined elementary functionalities, and for each elementary functionality there can be different concrete implementations. Then the elementary network elements can be combined in different ways to form a variety of neuron models. An example would be synaptic plasticity as elementary functionality and different types of plasticity as different concrete implementations implemented in different network elements. Moreover, with composition this can be done directly in run-time while constructing the NEVESIM network, without the need to recompile the C++ code.

The NEVESIM design methodology fully encourages composition through coupling of network elements, and having a fine granular structure of neuron and synapse models. Note that, however, in the NEVESIM framework we do not impose any conventions and specifications that define the way the network elements are coupled together to form the neuron and synapse models.

#### 2.1.3. Causal update links

A change in the network state of one network element due to an input spike on its input ports typically also requires a state update of the network elements it is coupled to. A typical example is the point neuron model that we discussed, composed of its input synapses as network elements that are coupled to the soma of the neuron implemented as another network element. In such a case when one of its input synapses receives a spike, after the synapse updates its state, the network element representing the soma should update the membrane potential of the neuron to reflect the change of the postsynaptic response of the synapse. Since in NEVESIM there is not a global clock which ensures that the state of all network elements will be updated at each time-step, the propagation of the state-updates through the coupled network elements should be handled explicitly by the NEVESIM framework.

For this purpose we introduced the concept of causal update link between network elements. A causal update link is a directed link defined with a triplet (src_id, dest_id, update_id) which connects two coupled network elements with IDs src_id and dest_id. The presence of such a causal update link means that whenever the network element src_id updates its state (due to a received spike or a causal update link from another network element), after that the network element dest_id should also perform an update of its state. The integer number update_id is an identifier which informs the network element dest_id what type of update it should perform. The state updates are carried out by the simulation engine based on the causal update link graphs.

**Figure 1:**
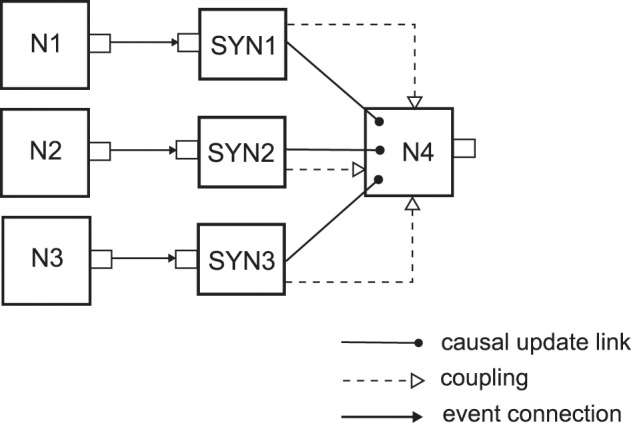
**NEVESIM network that implements a network structure of three neurons N1, N2 and N3 connecting to a fourth neuron N4**. The small rectangles on the right/left side of the network element represent their output/input ports, respectively. There can be three different types of relations between the network elements, a causal update link, a coupling, and an event connection, which are depicted as it is shown in the legend of the figure. One-way coupling of network elements is represented in the figure by a dashed line with one arrow at the end. The synapses SYN1, SYN2 and SYN3 have event connections at their input ports where they receive the spikes. There is one-way coupling from each synapse to the neuron because after it receives an input spike the synapse accesses the neuron and communicates to it the amplitude of the required instantaneous jump of the membrane potential due to the input spike, e.g. by invoking a function of the neuron network element. The neuron is not coupled back to the synapse as it receives everything that it needs from the synapse. The synapses also have a causal update link to the postsynaptic neuron, because after receiving the value of the instantaneous jump of the membrane potential from the synapse, the postsynaptic neuron should update its state (the value of the membrane potential), and additionally it should fire a spike and enter a refractory state if the new membrane potential crosses the threshold.

To better illustrate the usage of causal update links and coupling of network elements we will consider a simple example neuron model, an event-based implementation of a deterministic current-based leaky integrate-and-fire neuron model (definition can be found in Section 4.1.1 in Gerstner and Kistler, 2002) with Dirac-delta pulses as input currents caused by the input spikes. We assume that the neuron model is implemented such that its input synapses are separate network elements which are coupled to the network element of the neuron. Each synapse holds the value of the synaptic weight for that synaptic connection, and when an input spike arrives at the synapse at time *t*, the synapse informs the neuron that the membrane potential should instantly be changed by a value proportional to the synaptic weight. Then the neuron should update the state of the membrane potential *u_m_*(*t*_old_) from the last time of update *t*_old_ to the current time *t*, and add the value due to the input spike from the synapse. If the new value of the *u_m_*(*t*) crosses the threshold, then the neuron should spike. Figure 1 shows a small network structure with this event-based LIF neuron type where three neurons with IDs N1, N2 and N3 are connected to the N4 neuron, through the synapses SYN1, SYN2 and SYN3 respectively. This figure also introduces a diagram notation for the different possible relations that can exist between network elements, which we will use in the subsequent figures throughout the text. As it is shown, in order to ensure proper state update of the neuron N4 after an input spike, we need to create causal update links from each of the input synapses to the neuron N4.

Note that the causal update links just inform the simulation engine in what order it should call the state update operations, and creating a causal update link does not really perform any coupling of the network elements. The coupling is a separate operation that should be performed by the user with the provided interfaces of the network elements for coupling. In fact the three operations of creating event connections, creating causal update links and coupling of network elements can be seen as primitive operations in the NEVESIM framework. By combining these operations more complex and higher-level operations in the user interface can be defined. For example connecting two neurons with a synapse can be composed of three operations:

1. Create an event connection between the output port of the presynaptic neuron and the input port of the synapse,
2. Couple the synapse with the postsynaptic neuron, and
3. Create a causal update link between the synapse and the postsynaptic neuron.

We will describe in the following more precisely in what order the update operations should be executed in a given NEVESIM network. Let us assume that an event is emitted from a certain output port out_p of some network element. This output port can in general have many outgoing event connections with different delays. We group these outgoing connections based on their delay and consider one group of the outgoing connections that have the same delay equal to d, which we denote with Conn(out_p, d). We name the set of network elements that receive at least one event connection from Conn(out_p, d) as an *event target group* for the output port out_p and the delay d, or ETG(out_p, d). The ETG(out_p, d) network elements have the property that the event emitted from the output port out_p will be delivered exactly at the same time moment to each of them. This implies that the propagation of subsequent state updates from the network elements in ETG(out_p, d) to other linked network elements should be carried out concurrently, with the order of the updates determined by the graph of causal update links. More specifically, let V(out_p, d) be the set of network elements that can be reached through a path of causal update links originating from at least one of the network elements in ETG(out_p, d). Additionally, with G(out_p, d) we denote the directed acyclic graph that has V(out_p, d) as set of vertices, and all causal update links between them as edges. Then the update operations of the network elements should be executed in an order that is a correct topological ordering of the directed graph G(out_p, d). There can be many topological orderings of G(out_p, d), and the update operations can be performed in any of these orderings. As the graph of causal update links must be acyclic, if the user attempts to create a cycle of causal update links, NEVESIM detects the cycle and reports an error.

If a given network element E in G(out_p, d) has many incoming causal update links, these should ideally have the same value of the update identifier update_id. This value update_id will be given as an argument when the update operation of E is invoked. Update identifier values only become important in the case when a network element participates in two graphs G(out_p_1, d_1) and G(out_p_2, d_2) derived from two different event target groups. In such a case the network element can have different update_id values on the incoming links depending in the two different graphs, and execute different update operations depending on whether an event was delivered to the first or the second event target group. We will present an example of this later.

In the simulation engine the causal update links are implemented as an array of arrays (implemented as C++ STL vector data structures) where for each event target group ETG(out_p, d) there is an array of pairs (net_element_id, update_id) that represents a correct topological order of G(out_p, d). Thus, after an emitted event is delivered to the event target group ETG(out_p, d), in order to propagate the state updates one should simply iterate through the associated array of pairs. The used data structure is memory efficient as it stores just the minimum information required to correctly propagate the state updates. This is important as the number of causal update links for some networks can scale with the number of synapses which will make this data structure a significant portion of the memory footprint of the simulated neural network.

### 2.2. An overview of the architecture of NEVESIM

**Figure 2:**
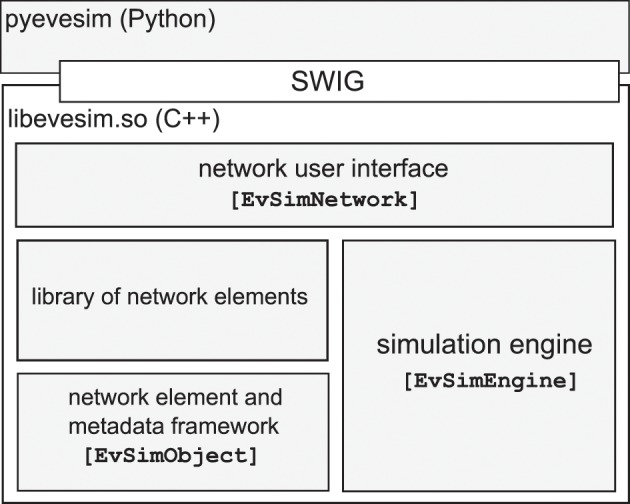
**The high-level architecture of the NEVESIM software package**.

The NEVESIM software package consists of the C++ library (libevesim.so) and a Python package (pyevesim) which wraps the user interface parts of the C++ library. There are four main components in the C++ library: the network user interface, the simulation engine, the abstract network element and metadata framework, and a library of already implemented and available network elements (See Figure 2).

The network user interface is implemented through the class EvSimNetwork that exposes to the user the set of all elementary operations in the NEVESIM conceptual framework that we discussed previously. Through the use of these elementary operations the user can construct various networks of network elements and control the simulation of the constructed networks.

The simulation engine, encapsulated in the class EvSimEngine, contains the algorithmic logic of the simulation strategy implemented in NEVESIM. As we pointed out before, NEVESIM employs an event-driven simulation scheme and the main execution loop of the simulation engine concerns the routing, scheduling and delivering events that are emitted by the network elements. The time ordering of the events is done with two priority queues: one for the scheduled events that are to be delivered in the future, and the second for pending future events that can be canceled. This is a standard way to implement event-driven simulation already previously used in other simulators (Brette et al., 2007; Taillefumier et al., 2012). In addition, the simulation engine is responsible for the propagation of state updates defined by the causal update links between coupled network elements.

The third component includes the abstract class EvSimObject from which all network element classes are derived, as well as a set of auxiliary classes that provide metadata and reflection capabilities for network element classes. With the metadata framework, for each network element a list of *fields* can be registered. A field is a member variable in the C++ class of the network element of a primitive data type (integer, float, double, char), which is usually either a parameter or a state variable in the model. If a member variable is registered as a field, then the metadata framework provides a set of facilities for accessing its value. For instance, one can retrieve the list of the names of the registered fields as strings, or get the physical address of a field for a specific created network element, by providing the name of the field as a string variable. This metadata field information is used in many places throughout the NEVESIM framework, as it enables configurability and access of the network elements on a meta-level, which for certain purposes is much more convenient as it achieves better generality with less code. It is also often used in the user interface, for example for setting up recording of fields during the simulation, or setting up modification of a field of one network element by another network element in the network. We will provide an example for this in Section 3.

NEVESIM also provides a library of already implemented network elements which contains neuron and synapse models, input generators, recorders, as well as other network elements that do some useful, more abstract processing. Apart from these readily available network elements, the NEVESIM framework allows the user to easily extend NEVESIM with custom network elements.

### 2.3. Composite neurons and synapses through C++ templates

Typically in the implemented model dynamics of neurons and synapses one can identify separate functional parts which interact together to realize the complete functionality. Moreover, there are neuron models which differ only in the implementation of one or a few such functional parts, whereas the rest of the implementation is the same. This suggests that it would be a good design strategy to encapsulate each functional part as a separate component through the utilization of some object-oriented construct, which will then enable building complete neuron models by putting together their constituent functional components. Such a fine-granularity design approach has the obvious advantage that each functional component can be reused in many different neuron models, which avoids duplication of the same code among neuron models. Thus, if one wants to modify the code of a specific functional part, one needs to make changes only at one place, instead of having to update the code of each neuron or synapse model that implements such functionality. Furthermore, if good mapping is achieved from the logical or functional components to object-oriented concepts, this makes the code more amenable to growth and extensions. Indeed, if a new feature or functionality is to be added and this can be achieved just by adding new lightweight components (classes), this is surely cleaner and simpler than having to modify and reorganize the code inside existing classes that are already in the codebase.

We already touched upon the design approach of having composite neurons and synapses in one of the previous subsections where this was achieved through object composition, or more precisely through coupled network elements in the NEVESIM framework. In this section we will introduce another design approach which in addition to using object-oriented constructs, also makes use of the generic programming capabilities of C++. We will explain the approach through an illustrative example that we describe in the following. The example concerns the implementation of several different types of synapse models as network elements. The synapse models that we want to implement differ in several aspects:

- The shape of the postsynaptic response can be either an exponentially decaying, double exponential or alpha shaped,
- the synapse can be either current based or conductance based,
- it can be a dynamic synapse, i.e., exhibit short-term plasticity or be a static synapse, and
- it can have either spike-timing-dependent plasticity, or rate-based Hebbian plasticity active.

We see that we have four functional parts in the synapse model, where each can have one of several different implementations. We should point out that the separation of the synapse dynamics into the functional parts presented above is not always possible, i.e., it is realizable only for some types of neuron models and simulation strategies. Nevertheless, it is a good and representative example for the approach that we want to present. In the design approach that we propose we organize the functional parts in a inheritance hierarchy of classes, where each functional part is implemented at a specific level of the hierarchy. Each additional level builds on and uses the functionality of the upper levels, and extends the functionality further with the logic of its corresponding functional part.

If we try to implement the class hierarchy with normal inheritance it becomes clear that it is not directly feasible to derive a class hierarchy that would encapsulate all different implementations and avoid code duplication. Namely, let us assume that we have three base cases for the different shape of the postsynaptic responses ExpPostSynResponse, DoubleExpPostSynResponse and AlphaPostSynResponse at the highest hierarchy level. In order to extend the exponential decaying postsynaptic response class with conductance-based functionality, i.e., add the second level, we can derive from ExpPostSynResponse a new class CondExpSynapse. But now if we want to do the same extension with the alpha shaped postsynaptic response and derive a new class CondAlphaSynapse from AlphaPostSynResponse, we should again implement the same conductance-based logic in CondAlphaSynapse that we already have in CondExpSynapse. From this it is evident that if we want to use inheritance to represent the different functionalities in this example there is a need to have a single class for the conductance-based logic for which we can somehow configure its base class it is inherited from, i.e., the type of the postsynaptic response. In our design approach we use C++ templates to enable this type of configuration of the base class. Templates in C++ are parameterized classes where the parameters are typenames, i.e., types of classes, that are used in the definition of the parameterized class. For example, let us assume that we want to implement a data structure, e.g., a FIFO queue. By using a C++ template, instead of providing a concrete data type for the elements stored in the queue, we can use a type parameter ElementType of the C++ template:

template<class ElementType>
class Queue {
   /* … the class implementation goes here … */
};


This enables us to use the same template implementation for instantiating queues of different data types, e.g., Queue<int>, Queue<double> or Queue<MyDataType>.

A useful property of C++ templates is that they can also have a parameter data type for the base class of the parameterized class. This is exactly the property that we use to achieve configurability of the base class for the classes that implement functionalities at lower levels in the inheritance hierarchy. In particular for the second level we have two parameterized templates: one for the conductance-based functionality and one for the current-based functionality, as follows:

template<class BaseClassType>
class ConductanceBasedSynapse : public BaseClassType {
          /* … the implementation … */
};
template<class BaseClassType>
class CurrentBasedSynapse : public BaseClassType {
         /* … the implementation … */
};


Similarly, for the third level we define two template classes, one for the dynamic synapse with short-term plasticity DynamicSynapse and one for the static synapse StaticSynapse, and accordingly for the fourth level two templates STDPSynapse and HebbianPlasticSynapse. All the template classes are parameterized by the type of the base class from which the template class inherits.

If we want to define now a current-based dynamic synapse which has an exponentially postsynaptic response and exhibits STDP plasticity, then we can combine the appropriate template classes at each level to define a new synapse type which implements the desired combination of functional parts:

typedef STDPSynapse<
            DynamicSynapse<
                 CurrentBasedSynapse<
                    ExpPostSynResponse>>>
                       ExpCurrBasedDynamicSTDPSynapse;


In this type definition with nested templates, in the innermost part we first define a class type CurrentBasedSynapse<ExpPostSynResponse> where the current based synapse CurrentBasedSynapse is inherited from ExpPostSynResponse. Then we use this class as a template parameter for the DynamicSynapse class which makes it a base class for the dynamic synapse, and we continue nesting for the other levels. In the same way we can define synapse types for all other possible combination of functional parts at the different inheritance levels. In order to be able to be used in such an inheritance hierarchy the template classes should certainly be compatible to one another. For example, all classes for the postsynaptic response at the top level should provide the same interface which is expected and used from the template classes at the lower levels.

## 3. Examples of NEVESIM network structures

### 3.1. Clock-driven network elements

As we described previously, the NEVESIM core simulation engine implements an event-driven simulation algorithm. Nevertheless, one of the goals during the NEVESIM development was to enable merging different simulation strategies in a single simulation framework, that allow simulation of heterogeneous networks of diverse neuron models and other network elements, some simulated in an event-driven, and some in a clock-driven fashion. It turns out that the algorithmic requirements for clock-driven simulation can be already achieved by utilizing the existing concepts in the framework. Indeed, what one needs to set up is a repeated communication of a signal to the clock-driven network element, usually at regular time intervals, so that it can advance its state to the time of the next clock tick. We implement such a signal with an additional network element that we call a clock.

In this implementation the output port 0 of the clock is connected to a set of clock-driven network elements via an event connection with delay 0. The clock also has a recurrent event connection from its output port 0 to its input port 0 with a delay equal to the time-step Δ*T* of the clock-driven simulation. Whenever it receives an event at its input port 0, the clock emits a new event at its output port 0. Hence, in such a setup the clock will output an event from port 0 after every time interval Δ*T*. We assume that the output port 0 of the clock is connected to an input port of a clock-driven network element which is dedicated for the time-step based update of its state, i.e., whenever the network element receives an event at this input port, the routine for advancing the state to the next time step is executed.

In the implementation above we considered only fixed time-step clock-driven simulation, however they can be readily extended to a variable time-step implementation. Namely, in NEVESIM it is possible for a network element to emit at time *t* an event with a future timestamp *t*_ev_ (*t*_ev_ > *t*) where *t*_ev_ can be arbitrarily chosen by the network element. Thus, in order to have a variable time-step, the clock can have a recurrent connection to itself with a delay 0, and emit at time t event *t* + Δ*T_k_* where Δ*T_k_* is the time-step used at the *k*-th iteration. This is sufficient, however, only in the case when the variable time-steps are known a priori, and can be implemented in the clock. A more common scenario is when the next time-step depends on the current state of the network element, for example when the neuron model uses an adaptive numerical integration algorithm with a variable time-step (Lytton and Hines, 2005). In that case the network element itself should decide and control when it will be activated next, i.e., it should be its own clock. This can be achieved with a recurrent event connection from one of the output ports of the network element to one of its input ports, dedicated for signaling the next time moment when the network element should be updated.

### 3.2. Description of the neural sampling models

In the next examples, we show how an efficient event-based implementation of the neural network models that implement neural sampling (Buesing et al., 2011) can be mapped to a network of interconnected network elements in NEVESIM. Before we continue with the examples, we first give a brief description of the neural sampling models.

The neural sampling networks are networks of spiking neurons that in their stationary stochastic dynamics perform MCMC sampling from a particular probability distribution. The neuron used in these networks is a stochastic point neuron model with a firing probability density at time *t* equal to $\rho (u)=\frac{1}{\tau }\exp\;(u(t))$ where *u* is the membrane potential of the neuron, and τ is a parameter which is also used in the definition of the postsynaptic responses and the refractory period of the neuron. The membrane potential is a weighted sum of the postsynaptic responses at the synaptic inputs *u*(*t*) = ∑_*i*_ *w_i_* ϵ_*i*_(*t*) where *w_i_* is the synaptic weight at the *i*-th synaptic input and *w_i_* ϵ_*i*_(*t*) is the postsynaptic response at the corresponding synapse. The postsynaptic response kernel ϵ_*i*_(*t*) is equal to 1 if there was a spike at the input in the time interval [*t* − τ, *t*], and otherwise equal to 0. After a spike, the neuron enters an absolute refractory period of duration equal to τ where it is silent. After the end of the refractory period it restores its normal stochastic firing defined above. The values of all state variables and parameters in this definition are in arbitrary units.

The connectivity of the neural network and the weights of its synapses are determined by the probability distribution it should sample from. We will describe this in more detail in Section 5.2. Notice that in the theoretically ideal neuron model defined above, the shape of the postsynaptic response is rectangular with a duration τ. Nevertheless, it has been shown in computer simulations that the error is not large and quite good approximations can be obtained when the network has biologically more realistic shapes of postsynaptic potentials. To explore such models, NEVESIM also supports simulation of networks with arbitrary shapes of postsynaptic potentials in the form of piecewise constant or piecewise linear kernels. Additionally, in the ideal model the delay of the synaptic connections should be 0, and also the τ parameter should be the same for all neurons in the network.

### 3.3. Synapses with shared postsynaptic responses

If we analyze the neural sampling models, we see that they lend themselves well for event-based implementation. Indeed, since the postsynaptic spike responses are rectangular, this implies that the membrane potential is a piecewise constant function. The membrane potential can change its value upon two types of events: when a new input spike arrives, or when an active postsynaptic response pulse caused by a previous spike ends (after time period τ). The new value remains constant until the next such event happens. Hence, after each input event, the membrane potential is constant, and we can calculate the time of the next spike under the assumption that the membrane potential remains constant until the neuron spikes. If the membrane potential changes in the meantime because of a new input spike, then the time of the next pending spike is again recalculated.

Another property of the neural sampling models that can be used to speed up the simulation is the fact that the evolution over time of the postsynaptic response ϵ_*i*_(*t*) is identical at each synapse originating from the same presynaptic neuron. Therefore, instead of implementing a postsynaptic response inside the network element of each synapse, we can have separate network elements encapsulating the dynamics of the postsynaptic responses, one for each neuron. In such a way the synapses that have the same presynaptic neuron can share the same postsynaptic response. A different, more commonly used speed-up strategy, based on a similar idea to reduce the integration of postsynaptic responses to one state variable per neuron, is to make the reduction at the postsynaptic neuron, i.e., to have one state variable for the total postsynaptic response of all input synapses of the postsynaptic neuron. This is possible when the postsynaptic responses have identical linear dynamics with the same time constants. However, although this common strategy works well for other typical shapes of postsynaptic responses (alpha, exponential, double exponential), for the rectangular shape it is not that efficient as the event-based simulation of the rectangular shape requires a delayed event for the end of the response to be scheduled in the event queue. Thus, calculating the total postsynaptic response at the postsynaptic neuron in a single state variable would imply scheduling a delayed event for each delivered spike rather than for each emitted spike. As the number of delivered spikes is much larger than the number of emitted spikes (each emitted spike generates many delivered spikes equal to the number of outgoing connections of the neuron that emitted the spike), it is clear that it is more efficient to use the approach where all synapses from the same presynaptic neuron have a shared postsynaptic response. But note that this approach is only feasible when all outgoing synaptic connections from the same neuron have the same delay, as only in this case the postsynaptic responses of the synapses have identical evolution over time.

To implement the shared postsynaptic response we use a network element of *spike response* type that has a rectangular response kernel. The spike response network elements are a type of network elements in NEVESIM that, in general terms, implement evolution of a variable with a dynamics that is determined by a sequence of events (spikes) and a response kernel. The dynamics of the variable is such that each new arrived spike triggers a response in the form of the response kernel which is either added to the value of the variable (in case of additive dynamics) or replaces the previous dynamics (in case of reset dynamics). Examples of variables that can be implemented with a network element of a spike response type are the input conductance or input current of a synapse, traces in implementations of STDP and other linear traces that occur in the dynamics of neuron models.

**Figure 3:**
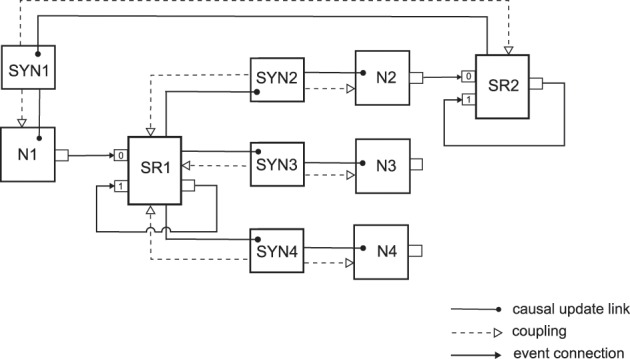
**An implementation with interconnected network elements of a neural network with four neurons and four synaptic connections**. The network represents a small part of a neural sampling network where synapses connecting from the same presynaptic neuron have shared postsynaptic response. In the network the neuron N1 connects to the neurons N2, N3 and N4 via the synapses SYN2, SYN3 and SYN4 respectively, and the neuron N2 connects back to neuron N1 through the synapse SYN1. See the text for details on the meaning of the existing connections and relations between the network elements.

Figure 3 illustrates the neural sampling implementation with NEVESIM network elements for a network with four neurons and four synaptic connections. As there are only two neurons that have outgoing synaptic connections, we have two spike responses as network elements, the spike response SR1 for the neuron N1, and SR2 for the neuron N2. Notice that, in addition to the input port 0 where they receive the spikes from the presynaptic neuron, the spike responses have an additional input port 1 and an output port which are connected through a recurrent event connection. The recurrent event connection is with a delay τ and the spike response network element uses it to signal to itself the time moment of the end of the rectangular pulse for the postsynaptic response. Since the neuron N1 has three outgoing synaptic connections, all three synapses are coupled to its associated spike response SR1, i.e., these synapses share the same spike response. The synapses are coupled, one-way only, to both their spike response and the postsynaptic neuron. The propagation of updates of states is carried out in the following order. When the spike response SR1 receives a spike on one of its input ports, 0 or 1, it recalculates the value of its postsynaptic response. Then afterward, as there are causal update links from SR1 to the synapses SYN2, SYN3 and SYN4, the state update operations of these synapses are called. In the state update operation the synapse first accesses the changed value of the postsynaptic response from the shared postsynaptic response network element (hence the one-way coupling to SR1). Then it communicates the changed value of the postsynaptic response multiplied by the synaptic weight to its postsynaptic neuron (hence the one-way coupling to the neuron). Finally, the causal update links from SYN2 to N2, from SYN3 to N3, and from SYN4 to N4 ensure that after the state update of any of the synapses, the state update operation of its postsynaptic neuron will be executed. The state update operation of the neuron updates the membrane potential to reflect the changed value of the postsynaptic response and then recalculates the time of its next spike.

The example showed that the concepts of the NEVESIM framework are versatile enough to implement a neural sampling network with shared postsynaptic responses between the synapses, which clearly optimizes the efficiency of the simulation. Indeed, this optimization achieves that the number of postsynaptic responses is equal to the number of neurons in the network, instead of being equal to the number of synapses. As the number of neurons is typically much smaller than the number of synapses, this can greatly reduce the simulation time of a neural network.

### 3.4. Synapses with plasticity

**Figure 4:**
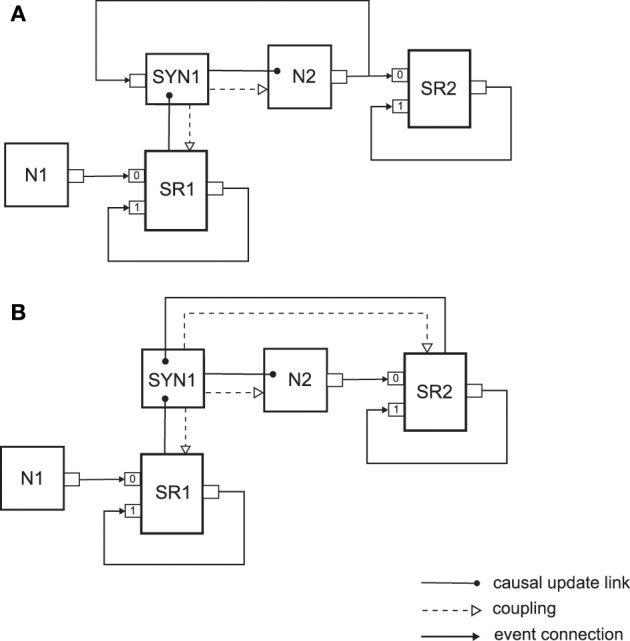
**Two types of plastic synapses that are used within neural sampling models**. **(A)** Implementation of a synapse with a shared postsynaptic response as a separate network element, and a synaptic plasticity mechanism that uses as input the times of the presynaptic and postsynaptic spikes. **(B)** Implementation of a synapse with a plasticity mechanism that uses as input the shared postsynaptic responses of the presynaptic and the postsynaptic neurons. See text for details.

The next two examples that we present show how synapses that exhibit synaptic plasticity can be embedded in a neural sampling network. In NEVESIM there are two types of plastic synapses used within neural sampling models for learning, which are shown in Figure 4. As it can be observed, both types of synapses assume implementation of a neural sampling network with synapses that have shared postsynaptic responses, which was already described above. They are, however, somewhat differently implemented with network elements. The first type of synapse (See Figure 4A) assumes a synaptic plasticity rule that depends on the presynaptic and postsynaptic spikes, and therefore it has an input port on which it receives the spikes of the postsynaptic neuron. Whenever there is a postsynaptic spike the synapse receives the spike on the input, and can update the synaptic weight according to the plasticity rule. If a presynaptic spike occurs, the synapse gets informed about that through the causal update link from the shared spike response. Within the state update of the synapse due to a presynaptic spike, in addition to communicating to the neuron the needed change in the membrane potential, the synapse can also apply the plasticity rule to modify the synaptic weight. The plasticity rule that is implemented by such a synapse can be some variation of a spike-timing-dependent plasticity rule. It is assumed here that the complete logic of the synaptic plasticity mechanism is implemented within the synapse network element. The second type of synapse depicted in Figure 4B instead of using the spikes, uses the spike responses of the presynaptic and the postsynaptic neurons as traces that are used within the plasticity rule. Therefore in this case the synapse network element is also coupled to the spike response SR2 of the postsynaptic neuron to be able to access the current value of its postsynaptic response. There is also a causal update link from the spike response SR2 to the synapse, so that the synapse gets informed when this spike response changes its amplitude. Thus, the state update function of the synapse gets called either through the causal update link from SR1 or through the causal update link from SR2. The synapse identifies where the state update was propagated from by the update id. Then, it can access the current value of the responses SR1 and SR2 as necessary according to the logic of the synaptic plasticity rule, and update the value of the synaptic weight (hence the one way coupling to SR1 and SR2). This type of implementation is convenient for plasticity rules where the traces in the rule have the same dynamics as the postsynaptic responses. In such a case, the speed efficiency of the implementation of the plasticity mechanism can be improved by not having to duplicate the implementation of the trace dynamics within the synapse, as it can be the case for the first type of plastic synapse implementation in Figure 4A.

## 4. Python interface

To create the Python interface of NEVESIM, the wrapping interface generator tool SWIG was used, which allows for rapid development of cross-language interfaces that interface C++ libraries to many different programming languages, including Python (Beazley, 2003). The Python interface has one to one mapping between the Python wrapper classes and the C++ classes in NEVESIM and preserves the same structure of classes as much as the semantics of the two languages allows. The benefit of this is that all instantiated network elements in the simulated network can be accessed and manipulated individually as Python objects in Python, in almost the same way as they can be manipulated in C++, which allows for full control of the NEVESIM functionality from within Python.

**Figure 5:**
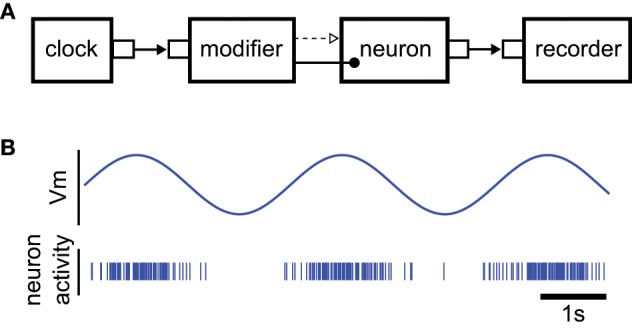
**Illustration of the example used to explain the main commands of the Python user interface**. **(A)** The diagram of the NEVESIM network that implements the simulation example. Since it modifies a field of the neuron the modifier is clearly coupled to the neuron, as indicated in the diagram, but as it can be observed, there is also a causal update link from the modifier to the neuron. The reason for this is that the neuron must update its state and recalculate the time of its next spike right after the value of its membrane potential was changed by the modifier. Additionally, there is an event connection from the clock to the modifier, which transmits the input events that trigger the update of the field, and a recorder network element connected to the output port of the neuron, which receives and stores the spikes that the neuron fires. **(B)** Depiction of the rate modulated firing of the neuron.

To illustrate the Python interface of NEVESIM, we consider an implementation of a very simple NEVESIM network, through which we present the commands in the Python interface that correspond to the basic concepts in the framework. The example implements a neuron that spikes stochastically with an instantaneous firing probability ρ(*t*) = 50 (*u*(*t*)), where *u*(*t*) is the membrane potential that evolves through time according to the sine function *u*(*t*) = 2 sin(*t*) − 1. After it spikes, the neuron enters an absolute refractory period of 1 ms duration, after which it restores its normal firing behavior according to ρ(*t*). The neuron type in NEVESIM that implements such stochastic firing is ExpPoissonNeuron. This neuron is typically instantiated with input synapses that modify the value of its membrane potential according to their postsynaptic responses induced by the input spikes. Here, however, we should modulate the membrane potential according to a predefined sine function. For that purpose we use a network element called modifier which has the functionality to repeatedly update the value of the membrane potential variable “Vm” which is registered as a field in the neuron. The field is updated according to a predefined array of values given to the modifier, such that every time the modifier receives an input event on input port 0, it performs an update of the value of the field with the next value in the array. In the example the input events to the modifier are generated with a clock network element which emits events at regular time intervals. The diagram of the NEVESIM network is given in Figure 5A.

The Python code that implements this simple NEVESIM network is given in Code Block 1. The construction of the network is started with construction of a network object of type EvSimNetwork in line 4. After creating the network object, we create the stochastic neuron in the network in line 6, by executing the method create of the network object, and giving as an argument a prototype neuron object of type ExpPoissonNeuron. The constructor of the prototype does not have any parameter values which means that default values for the parameters will be used. The provided prototype neuron object is then copied in order to create the actual neuron object within the NEVESIM network. Similar to this, in line 8 the clock network element of type EvSimRegularClock is created, which is setup to emit regular events with time interval of 0.001 s. With the next two statements in the code we create and setup the modifier network element which is specialized for modifying fields of double precision floating-point data type (hence the name DoubleVariableModifier for the modifier type). It accepts in the constructor four arguments: the network object, the ID of the network element with the field to be modified, the name of the field as a string variable (Vm in this case) and the predefined array of values sine_arr according to which the field is modified. During the construction of the modifier within the NEVESIM network also the coupling between the modifier and the neuron is setup. The coupling in this case assumes setting up the modifier to hold a handle (pointer) to the field Vm of the neuron. Apart from the coupling, we also setup a causal update link between the modifier and the neuron as required, by invoking the causalUpdateLink method of the network object with the IDs of the two network elements as arguments (line 14). After that, in line 16 an event connection is created from the output port 0 of the clock to the input port 0 of the modifier. This is accomplished with the connect method of the network object, which accepts as arguments the ID and port number of the source, the ID and port number of the destination of the event connection and the connection delay. At this point the only thing left to do regarding the construction is to setup the recording of the spikes of the neuron. As setting up a recording is a very common operation, there exist a convenient record method in the network object to do this. In line 18 we execute this method with the ID of the neuron as an argument, which creates a recorder and connects it to the output port of the neuron. The ID of the recorder network element is given as a return value of the method. The recorded spikes during the simulation can be retrieved, as shown in line 22, by first getting a handle of the recorder and then invoking its method getRecordedValues, which returns a numpy array of the recorded spike times. The handle of the recorder is obtained by executing the method getObject of the network object with the ID of the recorder as the argument.

**Code Block 1:**
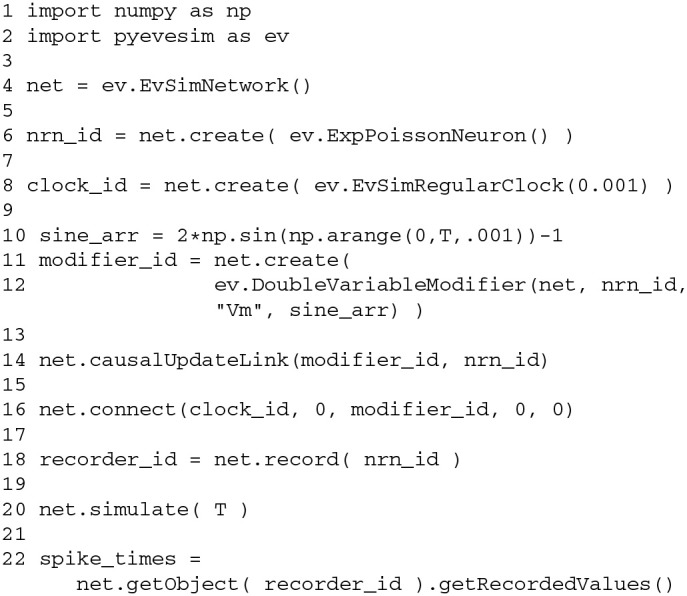
**A simple NEVESIM simulation example implemented in Python. See text for an explanation of the code**.

The retrieval of the recorded spikes after the simulation makes use of a useful feature of the Python interface, namely that we can access and manipulate every created network element inside the NEVESIM network as a Python object. This is made easily possible with SWIG by setting up automatic generation of Python wrapper classes for all network element classes in C++. Another feature of the NEVESIM architecture that is utilized in this example is the metadata framework, i.e., the possibility to access member variables in the C++ classes, which are registered as fields, by providing their name as a string. More specifically, in the example the modifier uses the metadata information of the neuron class to access the Vm member variable. The benefit of this approach is that the modifier class implementation is general in a sense that it can be used for modification of the value of any member variable of any network element type, which would otherwise be difficult to achieve in C++.

When running the code of the example we obtain a spiking activity of the neuron as shown in Figure 5B. The neuron produces rate modulated firing in phase with the sinusoidal curve of the membrane potential.

## 5. Simulation examples and performance evaluation

### 5.1. Benchmark model: random LIF network

As a first neural network example we simulated the standard benchmark 4 network model from Brette et al. (2007), which is a random connectivity, current-based LIF network with voltage jump synapses (i.e., the input currents due to input spikes are Dirac pulses). This type of network is very suitable to be simulated with an event-based algorithm as the differential equations can be easily solved analytically. Furthermore, as we already discussed in Section 2.1, the implementation of the neuron model nicely fits within the NEVESIM framework. We simulated the same network model as defined in Brette et al. (2007), with the same parameter values. The network consists of two populations of neurons, one excitatory and one inhibitory, forming 80% and 20% of the neurons, respectively, and the neurons are connected randomly using a connection probability of *p* = 0.02 (for other details of the model see Brette et al., 2007). As for the benchmark 4 the delays of the synaptic connections were not clearly specified in Brette et al. (2007), we set the the delay of all synaptic connections to be equal to 1 ms.

**Figure 6:**
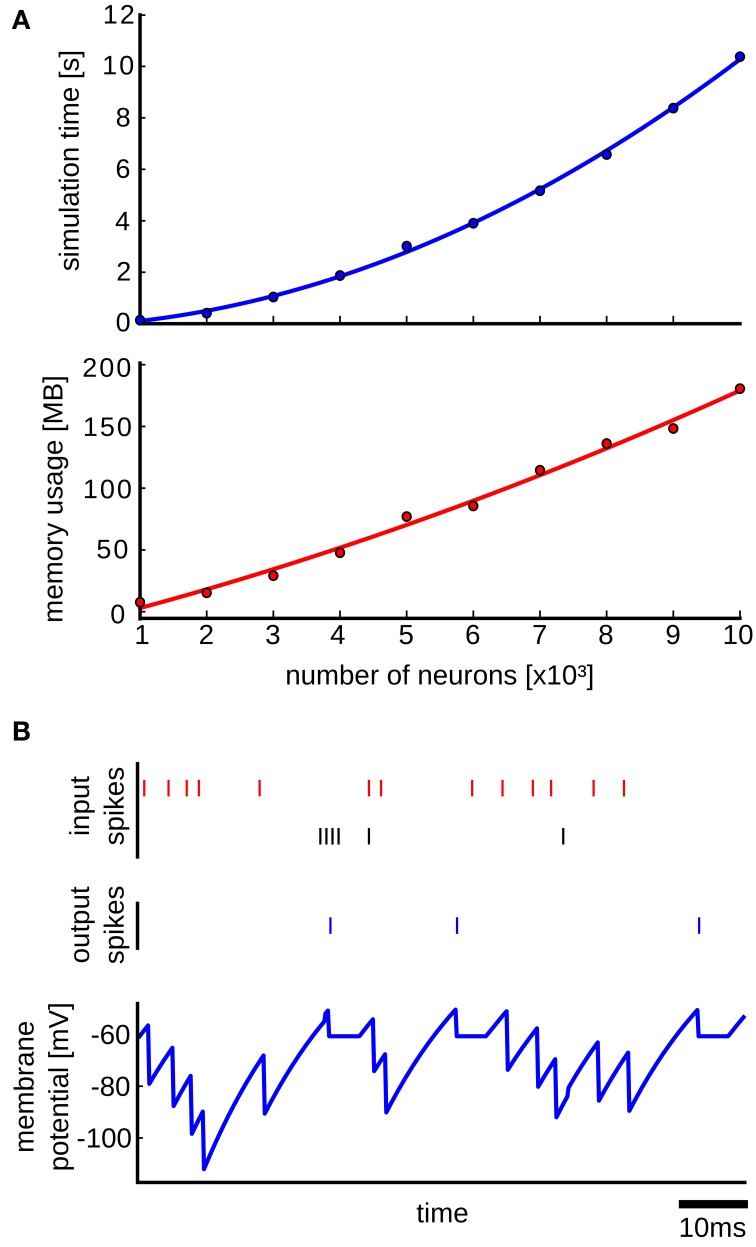
**Results from the simulation with the Benchmark 4 random LIF network from Brette et al. (2007)**. **(A)** Upper plot: simulation time as a function of the size of the network. Each point is an average over 10 simulation runs with different randomly generated networks of the same size. The standard deviation was too small and therefore it was not plotted. In each simulation run the network is simulated for 1s of biological time. The blue curve is a quadratic polynomial fit of the data points. Lower plot: same as in the upper plot but here the scaling of used memory with the size of the network is plotted. The red curve is a quadratic polynomial fit of the data points. **(B)** Simulation of a LIF neuron that receives input spikes from inhibitory (red spikes) and excitatory (black spikes) neurons and produces output spikes (blue) in response. The blue curve shows the membrane potential recorded in the NEVESIM simulation.

We performed simulations to test how the simulation time and the memory consumption scales with the size of the network. In each simulation run the network was simulated for 1 s of biological time. The simulations were done on a single core of a DUAL Xeon HEXA i7 3,46 GHz CPU with 96 GB RAM. The upper plot of Figure 6A shows the results, where it can be seen that the simulation time scales quadratically with the size of the network. This is expected, as the simulation time is mainly determined by the number of delivered spikes which scales as *O*(*r**pN*^2^) where *r* is the average firing rate of each neuron, *p* is the connection probability and *N* is the number of neurons in the network. We determined empirically the average firing rate *r* in simulations, and it turned out that it is approximately *r* ≈ 115 Hz for all network sizes. Thus, the number of delivered spikes indeed scales quadratically which results in quadratic scaling of the simulation time with the network size. In order to induce spontaneous network activity in this benchmark network, the resting potential was set in Brette et al. (2007) to be *E_L_* = −49 mV, which is above the threshold potential equal to −50 mV. This causes a high spontaneous firing rate of a neuron even without any input synaptic connections, which explains the high average firing rate in the benchmark network. The memory usage in the simulations scales also quadratically as is shown in the lower plot of Figure 6A. There are actually two main factors that contribute to the memory consumption. The first factor is the memory of the queue of scheduled events and the memory footprint of the network elements, which scales linearly with the network size. This is because we used two synapse network elements per neuron, which was possible since the weights of all excitatory synapses and all inhibitory synapses are the same in the model. The second factor is the memory used for the data structure for routing the events according to the event connections, which scales quadratically with the network size. As it can be seen in the lower plot of Figure 6A, the quadratic curve that fits the points is almost linear, suggesting that for the simulated network sizes the main memory consumption comes from the event queue and the network elements.

In addition to testing the performance, we also performed an accuracy test on a single LIF neuron that receives random presynaptic spikes from excitatory and inhibitory neurons, where we compared the recorded membrane potential of the neuron in NEVESIM, with the membrane potential obtained by analytically solving the differential equation. To record the membrane potential of the neuron in NEVESIM with a good resolution, we setup a *variable recorder* with a sampling rate of 5 kHz. Figure 6B shows the membrane potential from the NEVESIM simulation which matches very well the analytical solution. The mean squared difference between the two calculated membrane potentials was less than 10^−16^ (the analytical solution is not plotted as its plot is identical to the membrane potential from NEVESIM because of the small error).

### 5.2. Neural sampling networks

As we already pointed out, one marked feature of NEVESIM is its capability to efficiently simulate, through an exact event-driven simulation, neural models derived from a novel theoretical framework for computation with network of spiking neurons called neural sampling (Buesing et al., 2011; Pecevski et al., 2011; Habenschuss et al., 2013). The neural sampling theory gives a new perspective of how biological networks of neurons can perform probabilistic inference computations, by showing that, given certain assumptions, their stochastic dynamics can be interpreted as MCMC sampling. One of the values of this theoretical result is that it creates a link between many existing probabilistic computational models on a behavioral and cognitive level, and models of networks of spiking neurons which model brain computations on a neural level. Furthermore, neural sampling provides a bridge for porting a large body of useful results from the field of Machine Learning on MCMC sampling and stochastic computations with artificial neural networks, to the modeling research that uses networks of spiking neurons as more detailed, lower level models of brain computation. Thus, the capability to simulate neural sampling models brings an additional value to NEVESIM as a simulation tool. Indeed, it can be very useful for users that want to explore further the potential of the neural sampling theory and neural sampling models for elucidating various aspects of the organization of computation in networks of spiking neurons in the brain.

Let us consider a neural sampling network 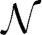 that in its stationary dynamics samples from a probability distribution with second-order interactions in the following form

(1) $$p(z)=\frac{1}{Z_{N}}\exp\left(\sum_{i<j}w_{ij}z_{i}z_{j}+\sum_{i}b_{i}\right)$$

where **z** = (*z*_1_, *z*_2_, …, *z*_*K*_) is a vector of binary random variables (RVs), *w_ij_* and *b_i_* are the parameters of the distribution and *Z_N_* is the normalization constant. The constructed network 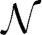 consists of *K* spiking neurons ν_1_, …, ν_*K*_, one for each RV in the distribution. Each neuron ν_*k*_ is a stochastic firing neuron as defined in Section 3.2 in the description of neural sampling models. According to the neural sampling theory, a sufficient condition for the network to sample from *p*(**z**) is that the membrane potential of neuron ν_*k*_ at time *t* is equal to $u_{k}(t)=b_{k}+\sum_{i=1}^{K}w_{ki}z_{i}(t)$. Here *b_k_* is the bias of the neuron, *w_ki_* is the strength of the synaptic connection from neuron ν_*i*_ to ν_*k*_, and *z_i_*(*t*) is the post-synaptic potential caused by a firing of the neuron ν_*i*_ that has value 1 during the time interval of duration τ after a spike of ν_*i*_, and otherwise value 0. If the network 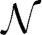 satisfies the sufficient condition, then its firing activity in the stationary regime generates, at any point in continuous time, a correct random sample from the distribution *p*(**z**). The samples are defined by the spikes of the neurons, by setting *z_k_*(*t*) = 1 if and only if the neuron ν_*k*_ has fired within the preceding time interval (*t* − τ, *t*] of length τ, and otherwise setting *z_k_*(*t*) = 0. A convenient property of neural sampling, similar to other stochastic systems in MCMC sampling in general, is that the same network 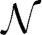 that samples from *p*(**z**) can also estimate marginal posterior distributions derived from *p*(**z**). Marginal posterior distributions are calculated in probabilistic inference tasks when we have concrete evidence about some of the RVs, and we want to estimate the probability of some of the unknown RVs given the evidence.

**Figure 7:**
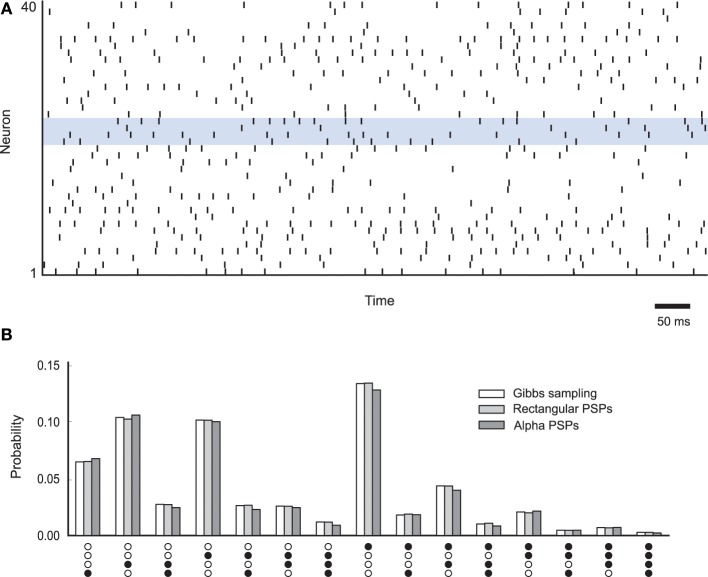
**NEVESIM simulation example with a spiking neural network sampling from the target distribution**. **(A)** Spiking activity of the neural network. The spikes of 4 neurons corresponding to the RVs for which the marginal distribution is calculated in **(B)** are plotted within a blue box. **(B)** Comparison between the marginal probability distribution *p*(*z*_20_,*z*_21_,*z*_22_,*z*_23_) over 4 RVs obtained with Gibbs sampling (white bars) and the estimated marginal probability distributions from the simulation of the neural network with rectangular PSPs (light gray bars) and the neural network with alpha shaped PSPs (dark gray bars). The bars show the probabilities of all possible assignment of values to the RVs *z*_20_, *z*_21_, *z*_22_ and *z*_23_, except the zero assignment (0,0,0,0).

In the following we describe a simulation example of a neural sampling network, in which we test whether the simulated network performs correct MCMC sampling from its underlying probability distribution. The neural network is composed of 40 neurons with absolute refractory period τ = 20 ms, similarly as in Buesing et al. (2011). The values for the *b_i_* and *w_ij_* parameters were drawn from a normal distribution with mean μ_*b*_ = −1.5 and standard deviation σ_*b*_ = 0.5, and μ_*w*_ = 0 and σ_*w*_ = 0.3, respectively. The spiking activity of this network is shown in Figure 7A. The probability distribution *p*(**z**) is estimated by counting the produced samples **z**(*t*) by the simulated network in continuous time. In other words, if we refer to **z**(*t*) as the state of the network at time *t*, then the probability of each state is estimated as the fraction of time the network spends in the particular state during the simulation. According to the neural sampling theory, the empirically estimated distribution should converge during the simulation to the target distribution *p*(**z**), which we have demonstrated in our simulation example (See Figure 7B).

The described neural sampling network can be implemented in NEVESIM with a few lines of code given in Code Block 2. In the code one assumes that the network consists of K neurons with refractory period of duration tau. We additionally assume that the biases of all neurons are given in one dimensional array b while the synapses variable is a list of dictionaries. Each dictionary in the list describes one synapse that is to be created in the network, and contains the ID of the presynaptic neuron, the ID of the postsynaptic neuron and the weight of the synapse. The network is simulated for T seconds. In the first line of the code we create a NEVESIM network object net which is an instance of the NeurSamplingNetwork class. In the next line we create *K* neurons via the create method of the net object. To simplify the code we use here *list comprehension* construct in Python which is a convenient way to create a Python list with a for loop. The return value of the statement in the second line is a Python list with the IDs of all created neurons. The used neuron type ExpPoissonNeuron to create the neurons implements a stochastic neuron according to the neuron definition in neural sampling (See Section 3). Its constructor accepts three arguments, where the first argument is the coefficient C in the firing rate function, in this case set to C = 1/tau, the second argument is the bias of the neuron set to b[i], and the third argument is the duration of the refractory period set to tau. After the second line we have a for loop which iterates over all synapses to be created. For each synapse the connect method of the net object is invoked in order to create a synaptic connection from the neuron with ID given by syn['pre'] to the neuron with ID given by syn['post'], by utilizing a so-called synapse factory object syn_factory (line 6). The utility of the synapse factory object is for the user to specify all traits of the synaptic connection that is to be created between the two neurons. In the particular case we specify that we want to have a composite synapse, which means that the synaptic connection will be composed of two network elements, the synapse network element and a separate network element for the spike response. For the synapse network element we use the type BasicActiveSynapse which is appropriate for neural sampling, and for the spike response we use the type ResetRectSpikeResponse which implements a rectangular shaped PSP. Note that the connect method of the NeurSamplingNetwork class differs from the basic connect method of the EvSimNetwork class, in that it implements a more complex functionality of connecting two neurons with a synapse, rather than just connecting two network elements with an event connection. Other than the rectangular shaped PSP, NEVESIM also has spike response classes that implement piecewise constant and piecewise linear shapes of responses, which can be used for approximating other arbitrary shapes of PSPs, like for example an alpha shape which we also used in our simulations. In this case, however, having higher precision of PSP shapes requires more segments in the spike response which comes at a price since it reduces the efficiency of the simulation. The piecewise constant and piecewise linear PSPs are intended to be used with the stochastic neuron model in neural sampling as well as other similar stochastic neuron models. The reason for this is that for this neuron model for many kernel functions typically used for the shape of the PSPs there is not an available analytical solution for the probability distribution of time of the next spike. For piecewise constant and piecewise linear PSPs, on the other hand, there exist a simple analytical solution for this distribution.

**Code Block 2:**
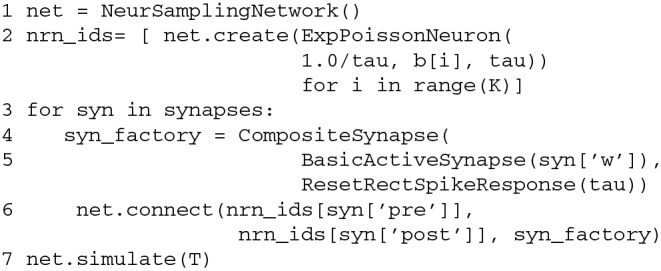
**A code snippet for generic implementation of a neural sampling network in NEVESIM**.

NeurSamplingNetwork class is derived from the EvSimNetwork class and implements additional methods for easier construction of neural sampling models, such as a connect method which is capable to connect two neurons with a synaptic connection. This type of operation is not available in EvSimNetwork, but can be achieved through the combination of methods that are available in EvSimNetwork. This is an example of another possible way to extend the general NEVESIM framework, apart from implementing custom network elements, i.e., by extending the network user interface with different more complex commands related to creation of user specific models.

**Figure 8:**
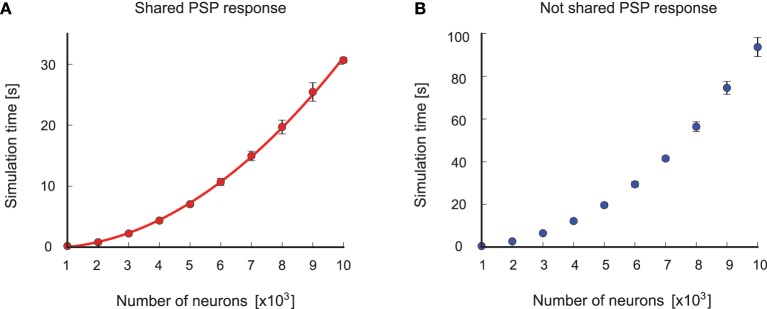
**Evaluation of the simulation performance for neural sampling networks**. **(A)** Scaling of simulation time with the size of the network for the implementation that used a shared postsynaptic response among the synapses with the same presynaptic neuron. Each point is an average simulation time, averaged over 10 simulation runs with different randomly generated networks of the same size. The vertical bars show the standard deviation. The solid line is a quadratic polynomial fit of the points. In each simulation run the network was simulated for 1 s of biological time. **(B)** The same as in **(A)** but for the implementation where the postsynaptic responses of the synapses are not shared.

In another set of simulations we evaluated the performance of NEVESIM for simulating neural sampling networks. For this purpose we simulated networks of different sizes which have random symmetric connectivity with *p* = 0.02 connection probability^3^. We used τ = 20 ms, rectangular PSP shape and set the biases of all neurons to *b* = −1. The synaptic weights were set to *w* = 0.3/(*p*(*N* − 1)) where *N* is the number of neurons in the network, and *p* is the connection probability. This achieved that the average sum of the input synaptic weights for each neuron is 0.3 for all network sizes, which resulted in the same average firing rate of the neurons independent of network size. In order to examine the speed up that is obtained when using the optimized implementation of neural sampling with shared postsynaptic responses for the synapses with the same presynaptic neuron, we performed the same simulations both with the optimized implementation and the implementation with each synapse having its own postsynaptic response network element. Figure 8 shows the results for both implementations. Comparison of Figure 8A and Figure 8B shows that using shared postsynaptic responses brings significant speed up for simulation of large networks. For the implementation with shared postsynaptic responses the simulation time should scale linearly with the number of delivered spikes, i.e., *T*(*N*) = *O*(*r**pN*^2^), similarly as for the benchmark 4 networks. For the second, not optimized implementation an additional factor for the simulation time is the number of scheduled events in the queue. As the reason why we simulated the same networks with the second implementation was just to demonstrate the speed up when using shared postsynaptic responses, we do not analyze further the complexity for this less efficient implementation.

## 6. Discussion

We presented in this article the NEVESIM simulation framework suitable for efficient event-driven simulation of spiking neural networks derived from the theory of neural sampling. Although the initial intention was to develop a simulator for neural sampling networks, the NEVESIM framework was designed to be general and extensible so that it can be extended for simulating also other types of neural systems with lightweight (simple) neural units. Furthermore, the concepts in the framework are not neural network specific and can potentially be utilized to define various types of abstract processing network nodes, which increases the flexibility of the simulator to be adapted to various kinds of simulated networks.

The central concept in the framework is the basic building block of a NEVESIM network, a network element. As we showed through examples, the concepts in the framework are capable to accommodate various types of neural models, and to support composite neurons and synapses, i.e., separation of the functional mechanisms of a neuron in different network elements. With this one can achieve better efficiency of the simulation when one has a mechanism encapsulated in a network element which is shared, i.e., used simultaneously by many network elements, as in the neural sampling model example with synapses sharing the same postsynaptic response. Another benefit of the approach is that if one already has a network element that implements some functional part in one neuron model, it can be reused in another neuron model that has an overall different implementation, but needs exactly the same functional part. Furthermore, another property of the framework is that it is designed with the goal to support simulation of heterogeneous neural (and other event-based) systems. Namely, every instantiated network element in a NEVESIM network can be of different type which is derived from the abstract concept of network element. This is a desirable feature as neural models with heterogeneous components are becoming more of a rule than an exception. Actually, the possibility to simulate heterogeneous systems is tightly connected with the support for composite neurons and synapses, since the composite neurons and synapses are composed of network elements that are of different type, i.e., encapsulate different functionalities.

Other than the approach that uses coupled network elements to break down the neuron and synapse models into smaller lightweight functional units, we outlined a second, complementary approach to achieve this through inheritance with C++ templates. In principle there is no general rule of thumb whether the first or the second approach is more appropriate to be used for a certain implementation. The choice depends largely on the current context and the particular requirements that one has, as well as on the design preferences of the developer. There are some authors, however, who argue that object composition should be favored over inheritance (See Gamma et al., 1994).

Compared to implementing the whole neuron model in a single network element, using the approach of separating the functional components of a neuron in multiple coupled network elements does introduce a certain overhead, both in memory consumption and execution speed. Regarding memory overhead, having multiple network elements implies usage of extra memory for each network element to store pointers to the network elements it is coupled to, and another extra pointer per network element to the table of virtual functions. The data structure that stores the causal update links between the coupled network elements also introduces an extra memory consumption. Regarding speed efficiency, it is more difficult to foresee the impact a certain implementation with multiple coupled network element would have on the execution speed, as it depends on the specific implementation, and also nowadays the compilers and processors introduce many optimizations that could make certain parts of the overhead irrelevant. In principle if there is a “heavy” interaction between the coupled network elements, and this interaction represents a performance critical part of the code, this could result in slower execution due to the fact that the network elements interact through function invocation, most of which are polymorphic, or through indirect access of their data members through pointers. Most of these extra function invocations and indirect accesses are avoided when everything is implemented a single network element. Nevertheless, a heavy interaction is usually an indication of a bad design, and one should aim for lightweight interaction where most of the processing is done not across, but within the network elements that are coupled. It is important to note that the NEVESIM framework does not force the user to implement the neuron model into coupled network elements in a specific way. Namely, if there is a use case when suitable memory usage or desired execution speed can not be achieved with coupled network elements, it is perfectly possible to have a single network element for the whole neuron model. It is even possible to have a single network element for a population of neurons as we will discuss later in Section 6.2 in context of specific optimizations for simulations of homogeneous networks. Nevertheless, as there are many use cases where separating the implementation into coupled network elements is beneficial, and improves the re-usability and flexibility of the code, the purpose of the framework is to provide basic conceptual tools that help the user to implement composite neuron models in a well organized way rather than using arbitrary ad-hoc solutions for each model.

In contrast to the coupled network elements approach, the template-based inheritance approach introduces virtually no overhead in execution speed and memory consumption. One of the reasons for that is that it does not have to use polymorphic (or virtual) functions for the interfaces of the classes. Namely, in object composition in order to achieve configurability in a sense that we can change the implementation of one functional unit with another without changing the code of the other functional units, we need to use abstract interfaces that are composed of polymorphic functions, and in C++ calling polymorphic functions is slightly more expensive than calling normal functions. With template-based inheritance we do not need polymorphic functions as the binding of functions is resolved in compile time instead of run-time. Moreover, in template-based inheritance the functions in the interfaces can be *inline* functions, so that the code can be further optimized by the compiler^4^. However, the template-based inheritance approach is not applicable in cases where the number and structure of the functional components within the neuron depends on the properties of the constructed network (i.e., connectivity, parameters or similar). For example, it is not possible to use it to separate each input synapse of the neuron as a separate functional component, which can easily be done with coupled network elements.

To date, in the built-in library of network elements of NEVESIM there are about 70 already implemented network element types. One subgroup of the network elements in the library consists of neuron and synapse models for different versions of neural networks that perform neural sampling, for example neurons with rectangular, piecewise constant or piecewise linear PSPs shapes, synapses with different plasticity mechanisms that are variations of the STDP rule in Nessler et al. (2013) and different intrinsic plasticity mechanisms. The library also contains the event-based implementation of current-based leaky integrate-and-fire neuron model with voltage jump synapses, used in the simulation example in this article that simulates the benchmark 4 network from Brette et al. (2007). Apart from the neuron and synapse models, there are also network elements that implement some useful basic functionality typically needed within the neural network simulations, for example input neurons that inject predefined spike patterns in the network, spike and signal recorders, clocks, variable modifiers, continuous time signal generators and others.

### 6.1. Related work

The growing use of networks of spiking neurons for biological modeling, as well as for implementation of spiking neuromorphic computing systems, has resulted in an increased interest in efficient algorithms for event-driven simulation of spiking neural networks. There have been a number of studies that made progress on that topic (Watts, 1993; Delorme et al., 1999; Mattia and Giudice, 2000; Lee and Farhat, 2001; Claverol et al., 2002; Marian et al., 2002; Connolly et al., 2003; Delorme and Thorpe, 2003; Makino, 2003; Reutimann et al., 2003; Rochel and Martinez, 2003; Brette, 2006; Ros et al., 2006; Rudolph and Destexhe, 2006; Brette, 2007; Tonnelier et al., 2007; D'Haene et al., 2008; Garrido et al., 2011; Rudolph-Lilith et al., 2012; Taillefumier et al., 2012) (See Brette et al., 2007 for a review). Concerning the part of the simulation algorithm for scheduling and delivering the events, various efficient solutions have been proposed based on different implementations of priority queues, like circular lists (Claverol et al., 2002), skip lists (Reutimann et al., 2003), heap trees (Lee and Farhat, 2001; Ros et al., 2006; Taillefumier et al., 2012) and quick-sort pools (Marian et al., 2002). If the connections delays are restricted to a finite set of values, it is also possible to use a set of FIFO queues (Mattia and Giudice, 2000). In Connolly et al. (2003) the authors provide a comparative performance evaluation of event-driven spiking neural network simulation algorithms using different implementations of priority queues. In NEVESIM we used two priority queues for handling the events, both implemented as heap trees, one variable length queue that holds the already emitted events and one constant length queue that holds the next pending event for each neuron. This event-handling strategy with two queues is well known (Brette et al., 2007) and already used in other implementations (Taillefumier et al., 2012). Apart from the event-handling algorithms, research work was also done on efficient event-driven simulation of more biologically realistic neuron models. In particular, efficient algorithms have been devised for a specific version of the spike response model (Makino, 2003), for current-based LIF neurons with exponentially decaying currents with different time constants (Brette, 2007; D'Haene et al., 2008), nonlinear quadratic IF neurons (Tonnelier et al., 2007) and conductance-based LIF neuron models (Rudolph and Destexhe, 2006; Brette, 2007; Rudolph-Lilith et al., 2012). Additionally, other studies have provided algorithms for stochastic LIF neuron models, either by analytically solving the probability distributions for the membrane potential and the next spike time of the neuron (Taillefumier et al., 2012) or by using precalculated look-up tables to represent these distributions (Reutimann et al., 2003). The approach that uses precalculated look-up tables to perform the calculations required for the neuron dynamics was also adopted in Ros et al. (2006) which enabled fast simulation of more complex neuron models in an event-driven framework for real-time applications.

The NEVESIM framework does not make any assumptions about the required properties of a neuron model that can be simulated, except that it communicates via spikes to other neurons. Hence, each of these discussed event-based neuron models can in principle be implemented within the framework and added to the library of network elements. How and whether the integration of the different state variables in the neuron should be decoupled in separate network elements depends on the specifics of the algorithm for the particular neuron model. Let us consider for example the conductance-based IF neuron model that has the same time constant for all input synaptic conductances and one state variable for the total input synaptic conductance. For this neuron model it is not necessary to calculate a spike response (input conductance) for each synapse in a separate network element representing the synapse, i.e., one can have a single spike response network element for the total input conductance coupled to the network element representing the neuron.

### 6.2 Hybrid event-driven and clock-driven simulation

Another aspect of the simulation framework that we analyzed was the possibility to incorporate clock-driven network elements within an intrinsically event-driven simulation system. We showed that this is feasible even without any changes of the simulation framework, and can be achieved with a special type of a network element which takes the role of a clock sending regular events, i.e., ticks, to the clock-driven network elements, so that they advance their state to the next time-step. Hence, from the viewpoint of software design, we see that the event-driven and clock-driven simulation strategies do not have conflicting demands and in principle it does not require a big effort to make them consistently coexist in the same simulation engine. It has already been argued that if one uses continuous spike-times that are not aligned to the time-step grid and performs exact time-step based integration of the neuron dynamics, the discrete-time simulation algorithm acquires event-driven features, and it is best to consider it as hybrid simulation algorithm, instead of either purely event-based or time-step based (Morrison et al., 2006; D'Haene et al., 2014). Related to this is the question of efficiency of time-driven vs. event-driven algorithms that has been recently extensively analyzed in Hanuschkin et al. (2010). There the authors show that for biologically realistic network connectivity, firing rates and range of delays for the synaptic connections, the time-driven algorithm with precise spike-times for linear neuron models developed in Morrison et al. (2006) is more efficient than the corresponding event-driven algorithm, while preserving the same numerical accuracy of the simulation. Furthermore, the algorithm from Morrison et al. (2006) has been generalized in Hanuschkin et al. (2010) to be applicable to any nonlinear neuron model with a threshold condition for spike generation, like for example the AdEx neuron model from Brette and Gerstner (2005).

In addition to the efficiency analysis of the developed simulation algorithms, the authors in Hanuschkin et al. (2010) also address the issue of implementing both event-driven and clock-driven neuron models in a single simulation framework. In particular, they outlined a generic approach for embedding an event-driven algorithms in a globally time-driven simulation framework. They used this approach to implement two event-driven algorithms for the purpose of comparing the simulation efficiency of those two algorithms to the time-driven algorithm with precise spike-times they developed. In this paper we also treated the question of hybrid simulation algorithms from the perspective of software framework design, where we showed that an extension is also possible in the other direction, i.e., that clock-driven neuron models can be embedded easily in a globally event-driven simulation framework. We expect that the oulined approaches from the NEVESIM framework are capable to provide the basic skeleton for implementation of the time-driven and event-driven algorithms described in Morrison et al. (2006); Hanuschkin et al. (2010). Another somewhat similar approach to NEVESIM for hybrid event and time-driven simulation was implemented in the EDLUT simulator (Garrido et al., 2011), where the authors introduce two different types of events scheduled in the event queue, one for the spikes and one for the state updates of all time-driven neuron models.

Closely related to the topics of discrete-time simulation algorithms is an optimization that can in some cases be achieved in such algorithms for homogeneous networks. Namely, one can use matrix and vector operations to speed up the computations for the numerical integration of the neuron dynamics. There already exist simulators implemented in a dynamic scripting language that achieve good performance by exploiting such a technique (Goodman and Brette, 2008), but one could possibly benefit from this idea also in C++, as there exist highly-optimized C++ libraries for matrix and vector operations. The NEVESIM framework is compatible with such an optimization, as a homogeneous population of neurons can be implemented in a single network element, with vector and matrix operations used in the implementation to make it more efficient. Being able to have a network element in NEVESIM with multiple input and multiple output ports makes it easy to implement such a network element and connect the population of neurons it contains to the rest of the network.

### 6.3. Python interface

The wrapping of the NEVESIM framework in the Python programming language brings many advantages to NEVESIM. Namely, it greatly improves the usability of the simulator, i.e., it simplifies the construction, simulation and analysis of neural models. Moreover, there are many high-quality software packages in Python for general scientific computing that can be utilized by the user together with the code for the neural model (Hunter, 2007; Oliphant, 2007; Pèrez and Granger, 2007). There are also useful software packages in Python for handling various accompanying tasks in a neural simulation project that are not handled by the simulator itself (Muller et al., 2009). This clearly brings great value to NEVESIM as it can be used together with a chain of powerful tools available in Python for handling different sub-tasks that arise in a modeling and simulation project. Regarding the generation of the Python interface, the used wrapper tool SWIG, with its support of automatic code generation of wrapper classes, made the whole process quite simple. Apart from simply running SWIG on the header files containing the definition of the C++ classes that needed to be wrapped, only a little extra effort was needed to make the Python interface functional. Although SWIG is a mature tool that handles quite well the mappings between corresponding programming constructs, concepts and functionalities between C++ and Python, as we have experience also with the wrapper tool Boost.Python (Abrahams and Grosse-Kunstleve, 2003) used for the simulator PCSIM (Pecevski et al., 2009), we must note that Boost.Python is to a certain degree more powerful than SWIG. Nevertheless, an important practical advantage that goes in favor of SWIG is that the compilation of the generated code is much faster than Boost.Python, and it was a suitable choice for NEVESIM as it certainly fulfills well its wrapping requirements.

### 6.4. Future work

Although the NEVESIM framework is designed to enable and support implementation of custom neurons and synapse models, the user still needs to have knowledge of C++ in order to be able to implement extensions. This could represent an entry barrier for potential users who want to use NEVESIM with their own implemented network elements, but do not have experience with C++. A typical approach to resolve this problem, adopted by many simulation software packages also outside of neuroscience, is to develop a higher-level language for specification of neuron and synapse models in a concise and intuitive way through text-based and/or visual diagrammatic specification. A special-purpose compiler can be then used to translate this specification to C++ code compatible with the NEVESIM framework. As NEVESIM would certainly benefit from adding such a specification language, it is one of the planned extensions that we want to implement in the future.

When extending NEVESIM for simulation of a specific type of neural systems, apart from the implementation of the needed network elements it is also important to implement a custom network interface to enable construction of the neural networks with suitable higher level operations. Depending on future requirements, the network user interfaces of NEVESIM will be extended to enable convenient construction and simulation of different types of neural systems and architectures, for example construction of balanced random networks. Additionally, operations for population and projection based constructions in the spirit of PyNN (Davison et al., 2009a) are also planned to be added, as well as support for PyNN.

The current implementation of the NEVESIM simulation engine performs a single-thread simulation, but in principle it is possible to extend it to a distributed simulation. The only constraint, in order to have an efficient parallelization, would be that the delay of event connections between network elements residing on different machines must be larger than some minimum delay value, where the minimum delay should be larger than 0. To provide parallel capabilities of NEVESIM our plan is to integrate NEVESIM with the PCSIM simulator (Pecevski et al., 2009) which already has a simulation engine that supports distributed simulation. Another emerging trend is to use GPUs for simulation of large-scale spiking neural networks, which provides very good scalability for much cheaper hardware in comparison to CPU-based distributed architectures (Fidjeland et al., 2009; Nageswaran et al., 2009; Thibeault et al., 2011; Minkovich et al., 2014). Using GPUs would be also an attractive option for NEVESIM to achieve good scalability. However, as one of the primary features of NEVESIM is to be user extensible and to enable simulation of heterogeneous networks that are composed of network elements of different types, this is in conflict with typical optimization techniques usually applied in GPU-based computing to achieve good performance and scalability (see discussion in Minkovich et al., 2014). Namely, GPU-based simulators for spiking neural networks are usually optimized for specific neuron and synapse types and typically simulate homogeneous networks. Thus, although in principle it is feasible to devise a GPU-based implementation of the NEVESIM simulation engine, it will most probably scale much worse than the specialized GPU-based neural simulators.

### 6.5. NEVESIM resources

More resources about NEVESIM can be found on its web page at http://sim.igi.tugraz.at/nevesim/. The web page contains installation instructions, as well as user manual with examples. NEVESIM is an open-source software project registered on sourceforge at http://sourceforge.net/projects/neuroeve from where the full source code can be downloaded.

### Conflict of interest statement

The authors declare that the research was conducted in the absence of any commercial or financial relationships that could be construed as a potential conflict of interest.

## Footnotes

^1^By exact simulation we mean a simulation algorithm which numerical precision is determined by the double floating point number representation.

^2^As to this end NEVESIM does not support parallel simulation, this is enough for network size ranges that can be simulated in one thread. If in the future there is a need to simulate larger networks, the network element ID data type can be easily changed to a 64-bit unsigned integer by modifying just one line of code.

^3^For each pair of neurons A and B, a binary value is drawn from a Bernoulli distribution with parameter *p* to decide whether they should be connected, and if the drawn value is 1 then A is connected to B and also B is connected to A.

^4^Inline functions are compiled by replacing every call of the function in the code with the function body, thus avoiding assembly call instructions and passing the function arguments through the stack, which can speed-up the execution of the critical parts of the code.
